# Yi Zeng: promoting good governance of artificial intelligence

**DOI:** 10.1093/nsr/nwaa255

**Published:** 2020-10-24

**Authors:** Hepeng Jia

**Affiliations:** Science Communication at Soochow University and a freelancing science writer for NSR

## Abstract

Artificial intelligence (AI) has developed quickly in recent years, with applications expanding from automatic driving and smart manufacturing to personal healthcare and algorithm-based social media utilization. During the COVID-19 pandemic, AI has played an essential role in identifying suspected infections, ensuring epidemic surveillance and quickening drug screening. However, many questions accompanied AI’s development. How to protect citizens’ privacy and national information security? What measures can help AI learn and practice good human behaviors and avoid unethical use of AI technologies? To answer these questions, Nation Science Review (NSR) interviewed Yi Zeng, Professor and Deputy Director at the Research Center for Brain-inspired Artificial Intelligence at the Institute of Automation, Chinese Academy of Sciences (CAS). He is a board member for the National Governance Committee of Next-Generation Artificial Intelligence affiliated to the Ministry of Science and Technology of China (MOST). Zeng is also in AI ethics expert groups at the World Health Organization and the United Nations Educational, Scientific, and Cultural Organization (UNESCO). He jointly led the drafting of Beijing AI Principles (2019) and the National Governance Principles of New Generation AI of China (GPNGAI, 2019).

**NSR:** AI development has brought many new challenges to human beings. For example, in a sense, the development of AI means robots replace human labor. How do we cope with this issue?

**Zeng:** AI certainly has a direct effect on future employment. This problem is embodied in two aspects. On the one hand, based on the current model, AI development is mostly based on data and integration. The data come from the practitioners whom AI is to replace. If the substitution trend is irreversible, then these practitioners should be given some reasonable rewards.

On the other hand, it is crucial to guide practitioners in the substitution process to follow the trend and reasonably adapt to the transition actively. For example, we can perhaps create community service, senior care or even family support as new future jobs.

Positive thinking from other countries has also inspired us. For example, the Brookings Institute in the USA published a report indicating that robots’ development makes us understand how we should redefine humanity and the meaning of work. The critical governance principle is fairly sharing. We should make social members involved in the AI development reasonably bear its costs and benefits [1]. Meanwhile, AI development should also overcome algorithm bias and learning error.

**NSR:** What is algorithm bias? In terms of AI governance, how should we deal with it?

**Zeng:** AI is based on algorithms so that it may be potentially associated with algorithm biases [2]. Because the algorithm is based on big data of social behaviors, it may implicitly reflect the social prejudice inherent in these behavioral data. AI models that learn these social prejudices may produce adverse effects on society.

To overcome the bias, we need to design measures to coordinate the AI system and society. We have to correct the mistakes displayed on the AI side. But the key is AI researchers should have an ethical awareness when designing and developing the technology. Now many researchers lack the understanding of ethics in their minds.

**Figure 1:**
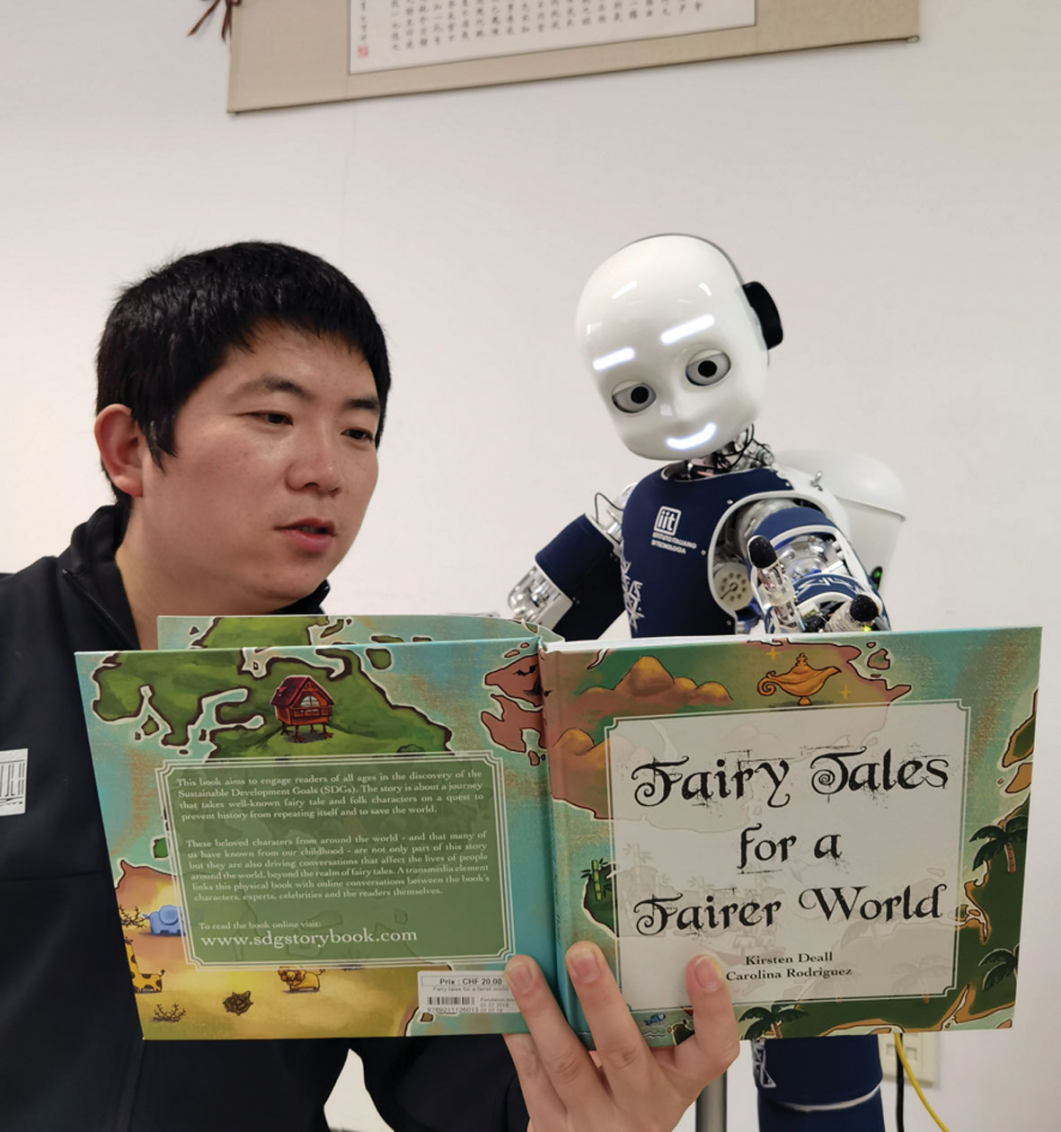
Prof. Yi Zeng and a Robot reading a book on global fairness *(Courtesy of Prof. Zeng).*

The GPNGAI published in 2019 claimed ‘fairness and justice’ as the most critical AI governance principle. GPNGAI promotes rights protection and equal opportunities. According to GPNGAI, prejudices and discriminations should be eliminated in data acquisition, algorithm design, technology development and product application. While being aware of algorithms’ inherent social biases, we can also develop an automatic algorithm

Because the algorithm is based on big data of social behaviors, it may implicitly reflect the social prejudice inherent in these behavioral data.

—Yi Zeng

to detect the potential biases. We should train AI professionals to avoid potential biases during the technology's design, development and deployment process.

**NSR:** GPNGAI is part of the national AI governance system. How about the AI governance situation in China?

**Zeng:** As a disruptive technology, AI has significant impacts on the future of all societies. We must ensure that disruptive technologies like AI are developing in a direction that is beneficial to society [3]. AI research and applications affect the community from different angles and at various degrees. Therefore, we must regulate not only AI researchers but also their products. AI models, applications and algorithms should comply with the moral and ethical norms of human society.

Many proposals on AI governance principles are specific to institutions, industries or a particular country. It would be vital to see how we can establish a framework that can be meaningful to different organizations, cultures and countries. GPNGAI released by MOST in June 2019 represents such a framework.

GPNGAI has eight central principles to govern AI development: harmony and human-friendliness, fairness and justice, inclusion and sharing, respect for privacy, safety and controllability, shared responsibility, open and collaboration, and agile governance. Agile governance indicates that AI governance should continuously adapt to technological development, optimize management mechanisms and engage with multi-stakeholders to improve governance.

Compared to other AI principles worldwide, GPNGAI particularly emphasizes using AI as an enabling technology to support the sustainable economic, social and environmental pillars of the United Nations Sustainable Development Goals. It promotes harmony, inclusion, sharing, openness and collaboration.

China is also developing a national governance system on AI. MOST has granted 11 AI innovative development pilot zones, including Beijing, Shanghai, Shenzhen, Tianjin and Hangzhou, to work on local versions of AI governance principles and regulations. As the first pilot zone, Beijing has been working on technical groundings of GPNGAI and released the Beijing AI Principles through the Beijing Academy of Artificial Intelligence. It is collaborating with partners such as CAS, Tsinghua and Peking universities and commercial companies like Megvii to set up a platform on data privacy and safe machine learning.

**NSR:** What is the learning error you mentioned above? Is there any ethical measure to prevent it? Have these elements been taken into account in the AI governance?

**Zeng:** Sometimes, the AI system has low adaptability. You can change the value of one of the pixels to subvert AI’s recognition results completely [4]. For example, a model algorithm may recognize some people with dark skin color as chimpanzees. In another example, Google's pattern recognition system may identify a 3D printed turtle as a gun. If a 10-year-old child holds this turtle, but the monitoring system recognizes it as a gun, there might be disastrous consequences.

Although it seems a question of recognition accuracy, this problem may bring extremely high costs. In AI research, we only see some typical examples in some specific industries. How to build ethical criteria to identify risks for such research is very important.

In GPNGAI, we stated that AI needs to be safe and secure and free from unintended harm. For safety issues and unintentional damage, we need to technically ensure that the AI models are designed to act in ways we desire and avoid adverse side effects. For security issues, we need to develop more robust and trustworthy models to confront aggression. AI industries are using many unreliable AI models. We need to set up a mechanism to evaluate AI services on possible safety and security issues before they are deployed.

Compared to other AI principles worldwide, GPNGAI [China's AI governance] particularly emphasizes using AI as an enabling technology to support the sustainable economic, social and environmental goals.

—Yi Zeng

**NSR:** AI has played a vital role in fighting the COVID-19 pandemic, particularly in East Asia. Meanwhile, it has raised a severe ethical challenge for privacy protection. Should the public give up their privacy in exchange for better protection?

**Zeng:** I don’t think the general public will give up their privacy because of the COVID-19 pandemic. We recently surveyed public opinion on facial recognition and public health. The general public argues that they can agree to have their private information gathered with an initial condition that the AI companies and the government departments must ensure their data's safety and security, open only to those who are necessary to be involved. They never want their private information leaked.

For example, health code has been very useful in monitoring the epidemic's transmission route. Again, the AI companies and governments need to ensure the safety and security of private information related to the code.

Indeed, the cultural aspect is crucial to assure AI’s successful use in epidemic prevention in China. In China, the self is built on relations with others and is denoted as a ‘relational self’. It is quite understandable in our culture that a single person's freedom cannot have a severe negative impact on other people's health. In traditional Chinese philosophy, we have ‘be in Harmony, yet be different’. We adopt this principle to coordinate AI governance among different cultures. But this traditional cultural value needs to be combined with personal information protection to ensure efficient and fair use of AI for public health.

Stressing international coordination is more valuable than claiming which culture is better to develop AI in applications such as disease control. For international coordination on AI principles, we need to have a set of commonly agreed values and beliefs. We should welcome diverse and different perspectives to supplement what we have decided. The UNESCO Global Recommendation of AI Ethics is along this line. People from different countries can extend understandings, complementary views and actions to realize the shared values and principles in AI governance.

**NSR:** For the implementation of S&T governance, we can see that bioethics has established codes of conduct and procedures, including mandatory institutional review boards (IRBs). Should we also develop a similar AI system, say, letting each research and development pass IRB review?

**Zeng:** I think, in theory, we should push forward the idea of an AI ethics committee that should provide service to AI education, academia and industry. We cannot guarantee that all experiments, products and services are without any problems, but we can help avoid many specific and unintended ethical risks. I propose to build AI ethics and governance public service platforms like AI Governance Online (http://www.ai-governance.online). The platforms should be used by anyone, not only for evaluations but also for education and training purposes.

On the other hand, any stakeholder in AI research, development, application and deployment, including scientists, practitioners, users and governments, is responsible for AI ethics and governance. More self-regulation from bottom-up is expected for AI research and development in academia, industry and other fields. Meanwhile, the government's top-down design and execution are essential.

Unlike in the biomedical field, where the treatment of and interaction with patients pave the way for bioethical rules, AI experts and practitioners are mostly trained as information technology engineers. Almost all of them have little training experience in ethics, not to mention AI ethics and governance. In the summer of 2018 at the University of Chinese Academy of Sciences, we started the course ‘Philosophy and Ethics of AI’, and now we will have the fourth version of the course. Over 80% of the participants are from engineering schools, and the rest 20% are from schools of humanity and management.

On the other hand, as we discussed above, the AI ethics committee is essential to keep AI for the proper use and avoid its potential misuse and abuse. It would be even more useful for the AI ethics committee members to interact with the technical team members within the same projects. We have already seen this happening in institutions, universities and companies in China.

## Notes

Hepeng Jia is a Professor of Science Communication at Soochow University and a freelancing science writer for NSR.
